# The 2023 Doyne Lecture—a cornea care system: evolution

**DOI:** 10.1038/s41433-024-03206-x

**Published:** 2024-07-08

**Authors:** Gullapalli N. Rao

**Affiliations:** L V Prasad Eye Institute, Banjara hills, Hyderabad, India

**Keywords:** Health care, Health services

## Abstract

Blindness and visual impairment affect the quality of life of the individual and their family members. Corneal opacities are a key cause of vision loss around the world, especially in low-income and middle-income countries (LMIC). Corneal blindness and vision loss impacts every age group, and the risk factors and the causes are also varied. Socio-economic factors also play a significant role in its prevalence. Preventing, treating, and managing corneal conditions in LMIC contexts can therefore be quite complex and challenging. A model of eye care delivery developed and refined over the past four decades, the L V Prasad Eye Institute’s cornea care system presents an example and a sense of hope. The model takes corneal care from world-class facilities in urban locations to rural locations, overcoming a variety of challenges. The initial breakthrough came with solving and ensuring a steady supply of corneal tissue. Then to training high-quality resources, building capacity, and investing in research that translates from the bench to the bedside. A variety of innovations, both in diagnosis and the operating theatre, have paved for improved outcomes that are tailored for the contexts in which this system operates. The institute envisions a future where its work further narrows the gap in eye care disparities and leads to life-changing impact in ophthalmic care of the cornea.

## Introduction

Blindness and visual impairment affect the quality of life of the individual and their family members. From a broader perspective, it impedes the socio-economic order and growth of the community and a nation. Global estimates show that a total of 596 million persons are blind and/or vision impaired [1]. An earlier study found that in 2015, about 3.2% of global blindness was due to corneal opacities [2]. The prevalence of blindness (presenting visual acuity <3/60 in the better eye) and of moderate to severe visual impairment (MSVI: presenting visual acuity <6/18 but ≥3/60 in the better eye) is highest in South Asia as compared with other regions of the world. South Asia includes 32.65% of the worldwide blind individuals, 28.25% of the worldwide individuals with MSVI, and 26.73% of the individuals with mild vision impairment (visual acuity between 6/12 and 6/18) [3]. In a recent meta-analysis on corneal blindness in Asia, the prevalence of MSVI and blindness due to corneal diseases in Asia was estimated at 0.38% (95% confidence interval (CI), 0.30–0.46), with the highest prevalence in India (0.88%;95%CI,0.38–1.57) and lowest in Sri Lanka (0.05%; 95%CI,0.00–0.11) [3–5]. In an earlier study from our Institute, the prevalence of corneal blindness was found to be 0.66% of the Indian population in at least one eye and included 0.10% prevalence of corneal blindness in both eyes and 0.56% in one eye [6].

## Corneal blindness

The causes of corneal blindness encompass a wide variety of infective and non-infective diseases that cause corneal scarring, which ultimately lead to functional blindness. Corneal involvement due to keratitis and corneal oedema are notably the common causes of corneal blindness in India [6–8]. Most of the reports on corneal blindness are in people 40 years or older; and so, there is an underrepresentation of corneal diseases in children and young adults. The major corneal causes of blindness in children include xerophthalmia, ophthalmia neonatorum, keratitis, and chemical injuries. The risk factors of higher prevalence of corneal blindness are related to poverty, water sanitation, climate, and geography. The age-standardised prevalence of blindness is higher for women as compared to men and likely explained by the differences in access to medical care facilities [9].

It is important to note that there is limited data on the global prevalence of corneal blindness. Similarly, corneal opacities require a systemic approach for prevention, treatment, and where required, transplantation and lifelong management. Overlaid with the complexities of care and management in low-income and middle-income contexts, managing the various causes of corneal blindness can be quite complex and challenging.

In this context, a model of eye care delivery rooted in such background, delivering high-quality corneal care, presents as a symbol of hope and as an example for providing care to those afflicted with blinding and seemingly intractable corneal diseases and conditions. The model is one of a “comprehensive cornea care system” including prevention, all components of care encompassing both medical and surgical care, followed by appropriate measures of vision rehabilitation. The model includes education programmes for ophthalmologists and other eye care personnel, research into areas of utmost significance, delivery models, and technological applications to enhance coverage of all parts of this system.

## A response: founding and expansion of LVPEI

The L. V. Prasad Eye Institute (LVPEI) was inaugurated in 1987 as a not-for-profit organisation with the vision of reconciling “Excellence with Equity” in Hyderabad, a metropolis in Southern India. The institute’s primary objective was to develop into a high-quality academic eye centre encompassing clinical care, education, and research. LVPEI’s central aim was to provide care to everyone who visited the institute, irrespective of their socio-economic background or ability to pay. The Institute then evolved to incorporate rehabilitation for those with irreversible vision loss as an additional component [10].

In 1995, the Institute expanded to the rural hinterlands, offering public health for eye care after recognising that care needs to be closer to the people in greatest need—the rural, agrarian communities with the highest burden of vision loss. This programme evolved into a pyramid model of eye care delivery over the next decade. The pyramid offers all levels of care: from primary to advanced tertiary services. The values motivating the pyramidal model include comprehensive eye care, a commitment to quality, a continuity of care through referrals to vertically integrated permanent facilities, and above all, compassionate care that is closer to the people’s doorstep without the patient worrying about expenses.

**Fig. 1:**
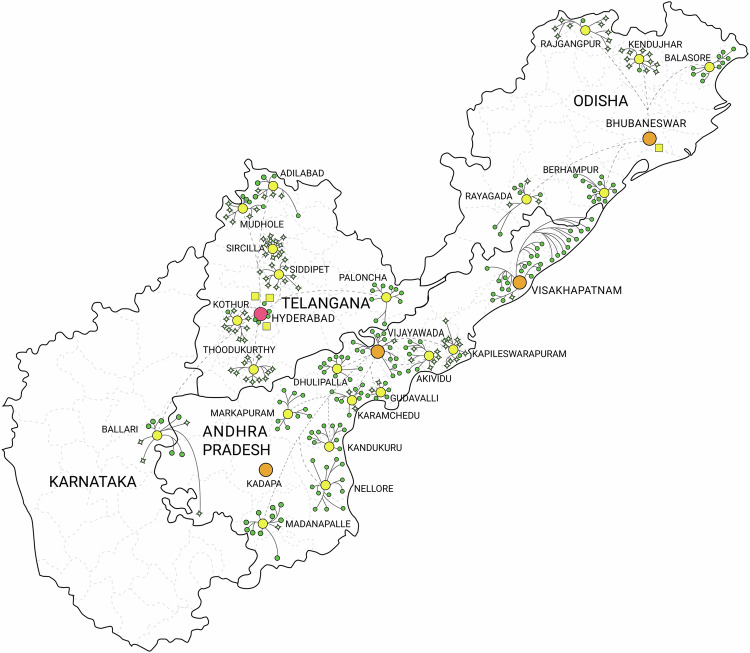
LVPEI’s 300 centres across the southern Indian states of Andhra Pradesh, Karnataka, Odisha, and Telangana.

The pyramid aims to provide appropriate care at every level that is available, affordable, and easily accessible to all people. This led to an effective and cost-efficient systems of eye care delivery. Over the past 37-odd years, this has proliferated into a network of 269 primary care vision centres, 26 secondary centres, three tertiary centres, and one Institute of Excellence offering advanced tertiary or quaternary care. This network of 300 centres predominantly covers four states of Southeast India, namely, Telangana, Andhra Pradesh, Karnataka, and Odisha (Fig. 1).

**Fig. 2:**
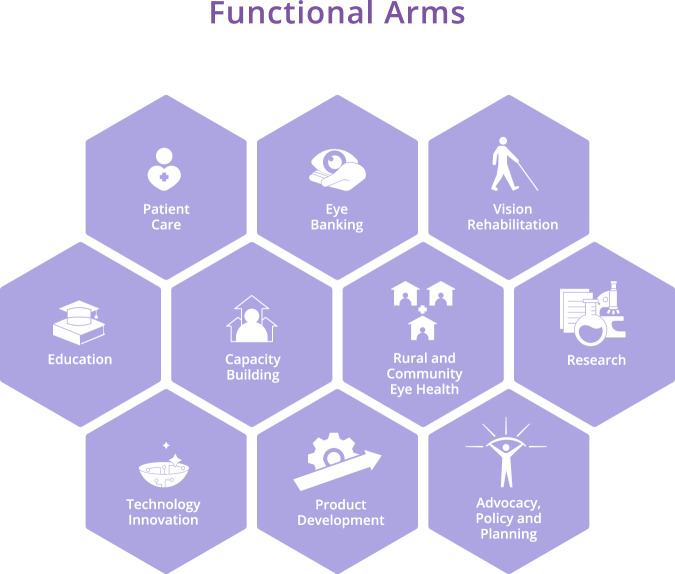
The 10 active arms of LVPEI.

The LVPEI network’s activities are covered through ten functional segments, which are interdigitated and work closely (Fig. 2). The network has evolved into a model of “Universal Eye Health” incorporating all its components—provision of eye health, prevention of vision impairment and preservation of sight, diagnosis, and treatment for restoration of sight, and rehabilitation for those with irreparable sight loss, offering them self-actualisation.

Corneal blindness has been one of the priority areas at LVPEI since inception. Most corneal blindness is preventable and this falls within the realm of public health programmes. Worldwide, the measures for promoting prevention of corneal blindness include Vitamin A supplementation, public education about trauma and infection, and the SAFE strategy for trachoma were prevalent [11, 12]. However, for those who develop a clinical condition, high-quality, hospital-based care becomes the critical difference between vision and sight loss.

## LVPEI’s cornea care system

A majority of the patients who come to LVPEI are already scarred by corneal disease, the main causes for which included prior trauma, infections, or post-operative complications [13]. So, the creation of a “Comprehensive Cornea Unit,” encompassing all critical components to address corneal conditions was a priority from LVPEI’s inception. The critical components of a high-quality infrastructure for medical and surgical care for all corneal diseases was created, with attention to necessary support services. Corneal care at LVPEI also included contact lens services and refractive surgery services with support from microbiology and ocular pathology laboratories. The establishment of dedicated microbiology services within the eye care model was critical to a systematic, primary approach to manage corneal infections effectively and reduce the severe disability that occurs as a sequela of the disease.

For clinical care, ophthalmologists who underwent corneal fellowship training were recruited or trained after recruitment in all aspects of corneal care through this approach. Both medical and surgical care are an integral part of this system. A large segment of corneal problems, when detected early, respond well to appropriate medical care or a combination of medical therapy and minor surgical intervention. Such care was integral to the cornea care system at LVPEI. Ocular surface disorders, dry eye, ectatic corneas, and other conditions constituted the major causes. LVPEI also cared for a large volume of children with corneal problems.

Many corneal conditions are non-responsive to medical therapy and result in a corneal scar and loss of vision. The only effective course in such a situation is surgical intervention, often in the form of corneal transplantation [14]. Successful outcomes of corneal transplants include graft clarity and visual rehabilitation. The key parameters governing the success of corneal transplantation are quality of donor cornea, the nature of recipient pathology (prognostic category), and issues related to post-operative care follow-up. The care system incorporated all these elements to achieve successful outcomes. However, the first and crucial enabler for all these services was a steady and reliable supply of transplant-grade corneas through an eye bank.

## Eye banking

In 1987, when the Institute began operations, eye banking in India was suboptimal. There were not enough facilities to meet the need, and those that did exist were not up to acceptable international standards. Corneal transplants carried a very high failure rate of due to a variety of factors. In the late 1980s, only about 7000 corneas were harvested and about 4000 transplant procedures were performed across India. Long waiting lists—running into years—for corneal transplantation was the norm in most institutions across the country.

Several myths circulated around the practices of eye donation and corneal transplantation, such as “nobody donates eyes in India”, and even “corneal transplants do not work on Indian eyes”. These myths sustained due to a lack of serious effort to raise public awareness about the positive aspects of eye donation and keratoplasty.

In the early years of corneal transplantation services at LVPEI, there were two well-trained cornea specialists who were dependent on corneas from an eye bank in Ahmedabad city in Western India and a few from US eye banks. This resulted in a waiting period of up to two years for these patients at LVPEI. Those who belonged to the non-paying category of patients (who are provided care at no cost) were admitted to the hospital several days in advance, to limit the burden of cost and logistics on them. Consequently, only 20 corneal transplants were performed during the first year. This was an unsustainable situation, and LVPEI made the decision to develop our own eye bank, adopting the highest international standards. Many sceptics dissuaded this initiative citing the high possibility of failure.

Despite the scepticism, LVPEI went ahead with its plans to develop an eye bank. The funding support from a local philanthropist provided much needed impetus for the project, while help was sought from US eye banking organisations for technical guidance. With an introduction from Dr Verinder Nirankari, Head of Cornea at the University of Maryland, the International Federation of Eye Banks (IFEB) in Baltimore, through its President Frederick Griffith, consented to be our technical partner. The design of the physical space, equipment, systems, and training of the first technicians were provided by IFEB. Thus, the first international standard eye bank in all of Asia and the developing world, the Ramayamma International eye bank (RIEB), was inaugurated at LVPEI in 1989. This was followed by continuous monitoring and evaluation by IFEB for many years, which helped in consolidating the quality of the eye bank [15]. Thus, local philanthropy and technical collaboration from established eye banking systems in the US were pivotal for the creation of the eye bank.

Following the establishment of the eye bank with the requisite standards, LVPEI turned its attention to increasing the number of cornea donors.

The adoption of Hospital Corneal Retrieval Programme (HCRP) from U.S. eye banks significantly accelerated the harvesting of corneas. At RIEB, the programme was initiated first in a large general hospital. HCRP became transformational and led to a significant increase in cornea retrieval. Within a year, the retrieval volumes tripled. The unreserved support from the leadership and administration at that first general hospital played a pivotal role in scaling up the programme. The success of HCRP at RIEB led to its adoption and replication in many other cities in India, leading to greater availability of donor corneas for transplantation [16].

This improved supply of corneas enabled a series of developments at RIEB. In the initial years, technicians would retrieve the full eyeball. Soon, eye bank technicians at RIEB began to produce the McCarey-Kaufman corneal preservation medium within the laboratory facility, which helped transition to retrieving just the corneal scleral rim. Over the years, HCRP expanded to multiple general hospitals, and RIEB built a modular clean room facility for preparing pre-cut corneas for lamellar procedures. This accelerated the progress of the work exponentially to keep pace with the changing techniques of keratoplasty. Till date, 103,844 corneas have been harvested at RIEB in Hyderabad. Over the past decade, three more eye banks were added to our eye bank network. Together, these eye banks harvested 10,077 corneas in the year 2022, with the eye bank in Hyderabad alone contributing 6322 tissues.

Aften an extensive evaluation of the retrieved corneas, they are distributed to cornea specialists within LVPEI and to other transplant centres across India.

In recent years, our eye bank network has been building capacity by creating new eye banks both within the country and internationally, in countries without eye banking. The strength of the eye bank services also helped medical and surgical care at LVPEI to blossom over the years.

## Corneal clinical services: medical care

High-quality infrastructure, the recruitment of well-trained cornea specialists for transplantation, and instituting a follow-up care system ensured a greater chance of transplant success. While the former two are addressed adequately by LVPEI (see below), follow-up care is far more complex. A significant proportion of patients come from distant locations, and the follow-up care required is often beyond the realm of the Institute, where the surgery is performed.

The nature of recipient pathology, namely the prognostic category, is another major determinant of transplant outcome. Consequently, proper understanding of pre-operative pathology is critical to prepare for any necessary modifications in the pre-operative surgical technique and post-operative care. A key requirement for this is high-quality training of ophthalmologists to make them into corneal specialists. LVPEI addressed good-quality training through a rigorous recruitment process of well-trained specialists, combined with additional internal training and regular upgradation of knowledge and skills through continuing education. LVPEI has also fostered an environment that enables the exchange of knowledge and nurtures talent by bringing in visiting professors with different perspectives and experiences. The faculty at the institute were provided with opportunities for advanced training at leading international centres, including participating in high quality international conferences.

That apart, a structured cornea fellowship was adopted at the institute to prepare trained cornea specialists that has led to a community of cornea alumni practising in different parts of India and elsewhere.

The ideal remedy for quality follow-up care is an ophthalmologist providing requisite care locally, access to medications, and finally, providing support infrastructure to achieve an optimal visual outcome. Each of these factors influences the longevity of graft clarity and visual rehabilitation of eyes that have benefited from keratoplasty. In many cases, contact lens fitting may be needed to correct post-keratoplasty astigmatism. One strategy employed was to familiarise many comprehensive ophthalmologists in the aspects of care for corneal transplants.

Several short-term and continuing education programmes were conducted periodically since 1988, which included two weeks observership programmes, and three-month, short-term fellowships in cornea care. Efforts were made to involve referring ophthalmologists in the continuity of clinical care of the patient. As for medications, availability of the right kind of medication, such as pure steroidal preparations (topical), were a problem in the initial years. This ameliorated over time by engaging with the ophthalmic industry, which responded positively.

## Contact lens

Similarly, contact lens manufacturers were encouraged to ensure supply of specialised types of lenses specifically needed for correcting post-keratoplasty refractive errors, such as high astigmatism. In fact, within a short time from its inception, LVPEI introduced a contact lens service to manage and correct post-keratoplasty astigmatism. Courses were conducted in the fitting and care of such lenses for both ophthalmologists and optometrists. The entry of major international corporations into India enhanced the availability of high-quality contact lenses. A key innovation in contact lens that is very effective in follow-up care contexts is the Boston scleral lens or Prosthetic Replacement of the Ocular Surface Ecosystem (PROSE) lenses [17]. These specialist lenses were available only in the U.S. and were very expensive. Despite these limitations, these lenses were procured for a few patients who could afford them. In 2017, LVPEI set up a unit to produce scleral and PROSE lenses licenced from the Boston Foundation for Sight (Needham Heights, Boston, USA). This initiative has helped reduce the cost of these lenses significantly and even enabled us to provide them at no cost to those who are economically less privileged.

## Corneal clinical services: surgical care

In the initial years, all corneal transplants performed at LVPEI were penetrating keratoplasties for both optical and therapeutic indications.

**Fig. 3:**
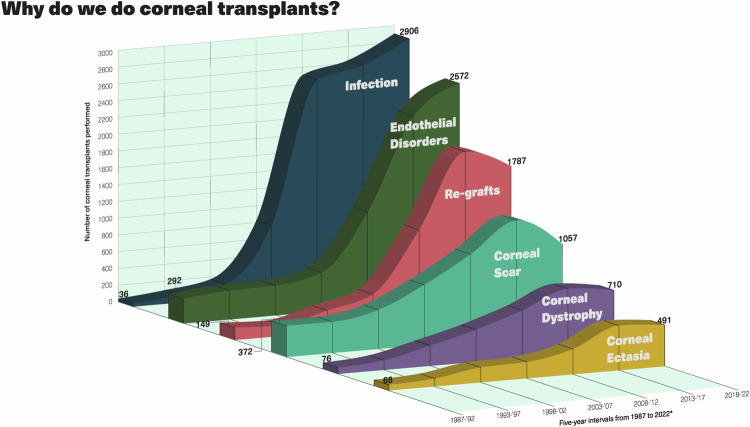
The major indications for a corneal transplant.

An analysis of patient data at LVPEI clearly demonstrates that the major indications for transplantation include active infection, corneal scarring secondary to infections/trauma/chemical injuries, endothelial diseases, degenerations, dystrophies, and developmental corneal diseases (Fig. 3). Geographically, LVPEI’s corneal transplant patients come from all parts of India and a minor subset from other countries, mainly the Middle East, Africa, and Asia.

**Fig. 4:**
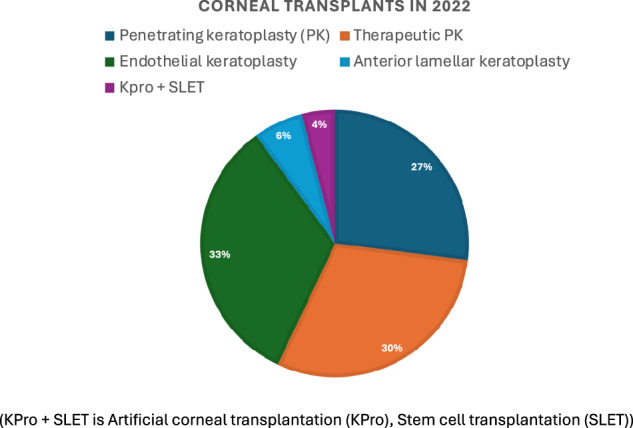
Types of corneal transplants performed at LVPEI and percentage breakup of volume.

In the initial years, all the transplants were for penetrating keratoplasty, both for optical and therapeutic indications, the procedure is now swaying towards lamellar procedures as shown in Fig. 4. Over these 35 years, the number of keratoplasties have increased in an accelerated manner. While only 10,000 transplants were done during the first 20 years, an additional 25,000 procedures were performed during the next 15 years. Of these, the last 10,000 procedures were performed in just four years. The total of over 35,000 procedures at our Hyderabad Centre with an annual addition of 2500 surgeries makes it, arguably, the largest volume corneal transplantation centre in the world.

**Fig. 5:**
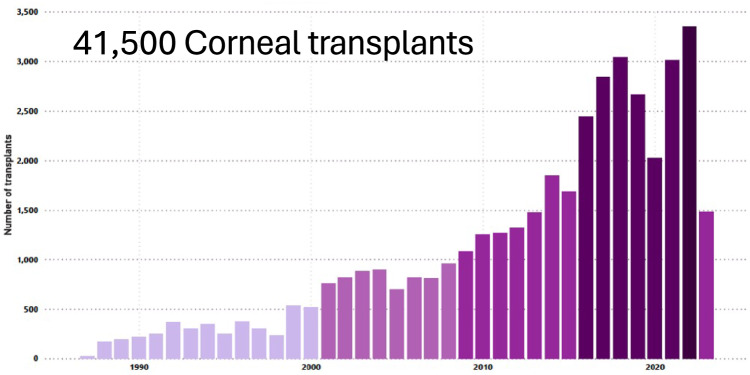
Total corneal transplants performed at LVPEI over 36 years.

Across the LVPEI network, nearly 50,000 corneal transplants have been performed since inception. Figure 5 illustrates the steady growth in volume over the years across the LVPEI network. While 56.3% of these patients paid for their services, 43.7% received these at no cost. Over 63.99% of our patients were male and 36.01% were female.

## Surgical innovation

LVPEI’s cornea care system is not about volume alone, investments and efforts towards surgical innovation are key to the system’s enduring success. Take the case of end-stage and recalcitrant corneal diseases that are not amenable to conventional transplantation. Many such cases need alternative surgical management with keratoprosthesis, including the commonly used Boston Keratoprosthesis [18]. At LVPEI, around 647 procedures have been performed for various indications that included high risk keratoplasty, multiple failed grafts, and eyes with severe ocular surface diseases. In cases with completely dry eyes and severe end-stage keratopathy and blindness, an LVPEI innovation, LVP keratoprosthesis (LVP Kpro) has shown encouraging results [19]. An LVP Kpro is a modification of the device that has been used for eyes that have severe grade dermalisation of the ocular surface. Our group is also the first to describe the process of a novel simplified surgical technique for the treatment of unilateral limbal stem cell deficiency - The Simple limbal epithelial transplantation (SLET) [20]. SLET has been reported as a cost-effective strategy compared to cultured limbal transplants [21] and is useful even when the culture facility is not available [22].

In children, a novel surgical approach, the Selective Endothelialectomy in Peters Anomaly [23] has been described as an alternative to paediatric keratoplasty in the management of anterior segment dysgenesis. Additionally, several clinical guidelines and strategies have been elucidated in the diagnosis and management of several clinical conditions, such as keratitis [24], corneal hydrops [25], and corneal dystrophies [26].

## Corneal care in rural settings

Universal eye health is made possible by focusing on the availability, accessibility, and affordability of eye care. This principle holds true for corneal care services as well, and it is important that such care is closer to the people who need it the most. To make this a reality, LVPEI initiated corneal transplantation services in our rural, secondary centres through a new strategy [27]. The donor cornea is provided by the eye bank at a tertiary centre and the surgical procedure is performed by one of our cornea specialists who travels to the secondary centres periodically. Post-surgery follow-up care is provided by the comprehensive ophthalmologist trained in the care of corneal transplants and works at that secondary centre. The comprehensive ophthalmologist has access to additional support through telemedicine with a specialist at the tertiary centre, if required. The infrastructure for all this exists at LVPEI secondary centres. The basis for this initiative was to bring this service closer to where most problems exist, namely, rural areas.

The data suggests that rate of compliance to follow-up visits is higher among these patients when compared to the tertiary care centre because of geographic proximity and ease of travel. In fact, evidence points to the fact that a vast majority of patients can benefit from early access to corneal care through LVPEI primary and secondary centres, leading to better visual outcomes and significant cost savings for the patients [28].

## Innovation and technology in cornea care

One of the engines that have enabled this remarkable reach for corneal care at LVPEI has been our longstanding investments in appropriate technology. The Institute’s Centre for Technology and Innovation, several new ideas, such as remote care with teleophthalmology, were conceptualised, and some of these led to products of great value in enhancing care for cornea patients. One example would be “Grabi”, a simple camera support attachment to a smart phone coupled with an application (app) to share the resultant photo of a cornea. The attachment helps the patient or a family member take an image of the cornea, which can then be transferred to the cornea specialist for consultation. This device proved to be of immense value during the COVID-19 pandemic for follow-up care for corneal transplants, and other corneal conditions. It also minimised the number of follow-up visits, thereby adding to the patients’ convenience and comfort. Grabi is now routine practice at LVPEI, where doctors prescribe this device to all patients who undergo corneal transplants at our Institute.

Along with the app, LVPEI doctors also leverage teleophthalmology to care for and manage corneal diseases at the primary level of care. All LVPEI primary care ‘vision centres’ are linked to the command centre in the tertiary centre where a consultant is available to provide an opinion [29]. The teams use a triage system, which helps the consultant determine the treatment that can be provided right at the vision centre or opt for a referral to a secondary or tertiary centre. This system enables an early diagnosis and prompt referral when needed and avoids unnecessary visits to the tertiary centres, reducing the burden of costs of travel in addition to convenience.

Our data showed that 84% of patients could be managed at the primary care level through this online consultation. Teleophthalmology and the Grabi app provide substantial financial savings for our patients, and the overall carbon footprint of patient travel also reduces [29]. In a recent study we found that, over a three-month period, online consultations resulted in the saving of 176.6 kg CO_2_/person and 1666 km/person for deferred tertiary centre visits enabled by our telemedicine approach. The patients saw an average savings in travel cost to a secondary centre of INR 370 (USD 4.6) and INR 8339 (USD 104) to a tertiary care centre.

## Education

The other engine that supports LVPEI’s corneal care are our education programmes. LVPEI’s vision for cornea training focusses on capacity building across the country to train ophthalmologists who can tackle the myriad of corneal conditions they see in their clinics. The training includes a three-tier programme: post-residency fellowships, both long and short, and observerships. Long-term fellowship was of one year duration initially and was converted to two-year period some years later. This is to build a centre of well-trained cornea specialists for the entire country and other developing countries. The short-term fellowship is for three months. It is for those who are practicing ophthalmologists, including those who provide care for corneal conditions, despite never taking formal training in cornea. The two-week observership is to expose ophthalmologists on the proper care for infections, common corneal problems, and post-operative care for transplants.

In addition, LVPEI offers continuing medical education through various modalities. Cornea alumni are spread throughout India, constituting about half of all trained cornea specialists in the country. Globally, LVPEI’s alumni network extends to many countries, particularly the developing countries. Many of these have become catalysts for better quality of care for corneal problems in their geographies and lead high-quality corneal programmes there. Our fellowship programme is now one of the biggest in the world. Similarly, education programmes are offered to all eye care professionals in the cornea value chain, such as eye banking professionals and contact lens dispensing optometrists.

## Capacity building

Another active functional area at LVPEI is building the capacity of other hospitals in developing countries that offer corneal services. Many national and international programs have benefited from LVPEI’s capacity building efforts. About 8–10 eye care organisations in India were provided technical support in setting up their own corneal units. Internationally, countries like Bangladesh, China, Indonesia, Nepal, Bhutan, Myanmar, a few sub-Saharan African countries, and countries in Latin America were given technical support to setup cornea care systems. Support included getting their teams trained in surgeries and management of operating rooms and many other areas. Alumni from around the world collaborate with LVPEI in enhancing the care of corneal conditions in their countries adding to the larger impact of our work. The alumni network is spread across the country and abroad. In 2017, LVPEI helped setup the Liberia Eye Center in Monrovia, Liberia’s capital. The centre was formally inaugurated by the then President of Liberia and Nobel Peace Prize winner, Her Excellency Dr Ellen Johnson Sirleaf, and is working to develop infrastructure and human resources to meet the needs of Western Africa. Education activities such as annual diploma or certificate courses in the hospital management, training of OR nurses, and initiation of the ophthalmic nurse training programmes are also run regularly.

## Collaborations

A key source of LVPEI’s success in growing and sustaining its vibrant role in cornea care has been the many collaborations from around the world. The collaborations range from education to research activities. These include world-renown centres of care in the US, Europe, and Australia. A new development, especially after the pandemic, has been large, peer-to-peer online networks of care providers. LVPEI is active, for example, in Project Extension for Community Health Outcomes (ECHO) [30], which has an ‘all teach, all learn’ approach to sharing clinical experiences. This project brings together practitioners from many countries to discuss anonymised, real clinical cases that enriches clinical practice.

Similarly, LVPEI works with a number of eye care funding organisations to help them build capacity for their referral hospitals and networks.

## Cornea research

Research has been an integral activity at LVPEI from its founding, and one of the first areas of focus was cornea research. These research interests include stem cell biology and transplantation, ocular surface, dry eye, infections, eye banking, and corneal transplantation, paediatric corneal problems, other corneal dystrophies, keratoconus, the endothelium, and immunology. These research interests covered most aspects of basic, clinical, translational, and public health research, contributing to the better understanding of some problems and finding new solutions or improving existing ones for management with better outcomes. Over the years, LVPEI faculty have secured competitive grants from the government, funding agencies as well as private philanthropic funding. Over 700 peer reviewed publications since 2002, several hundred presentations at international meetings, editorial board positions, invited lectures, and sustained research funding make for an excellent track record.

LVPEI’s major contributions have been in the areas of stem cell biology [21] and ocular surface [31, 32], corneal infections [24, 33], eye banking and corneal transplantation [15, 16, 34], contact lenses, and public health related to corneal conditions [35, 36].

## Future directions

Looking ahead, LVPEI’s cornea care system aims to continue expanding its horizons. The institute envisions a future where its work further narrows the gap in eye care disparities and leads to life-changing impact in ophthalmic care of the cornea. The commitment to LVPEI’s core values remains steadfast, as does the institute’s dedication to improving the lives of those affected by vision impairment. The new frontiers for LVPEI include big data powered AI to improve both research output, patient experience, ocular pharmacology, and therapeutics among others. Leveraging the power of the electronic medical records system, which has been the engine driving clinical documentation at LVPEI over the past decade, the institute is now embarking on mining insights that improve prognostic and diagnostic accuracy for the benefit of the patients.

The Cornea care system pioneered at LVPEI has made a mark for itself and has set the standard of care for a range of disease contexts. The system’s impact on ophthalmology extends far beyond its laboratories and clinics. Its research, steeped in LVPEI’s values of ‘patient-first’, excellence, equity, integrity, and togetherness, has set a benchmark for what is possible even in the Global South. As the institute looks to the future, it holds the promise of continued innovation and advancement in eye health, remaining a beacon of hope and excellence in the field [37].

## Conclusion

**Fig. 6:**
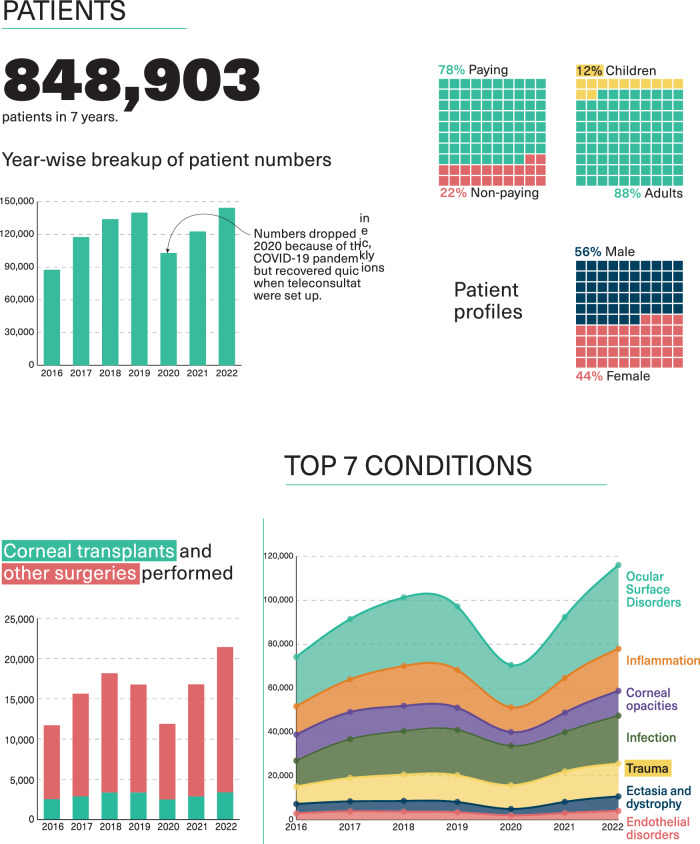

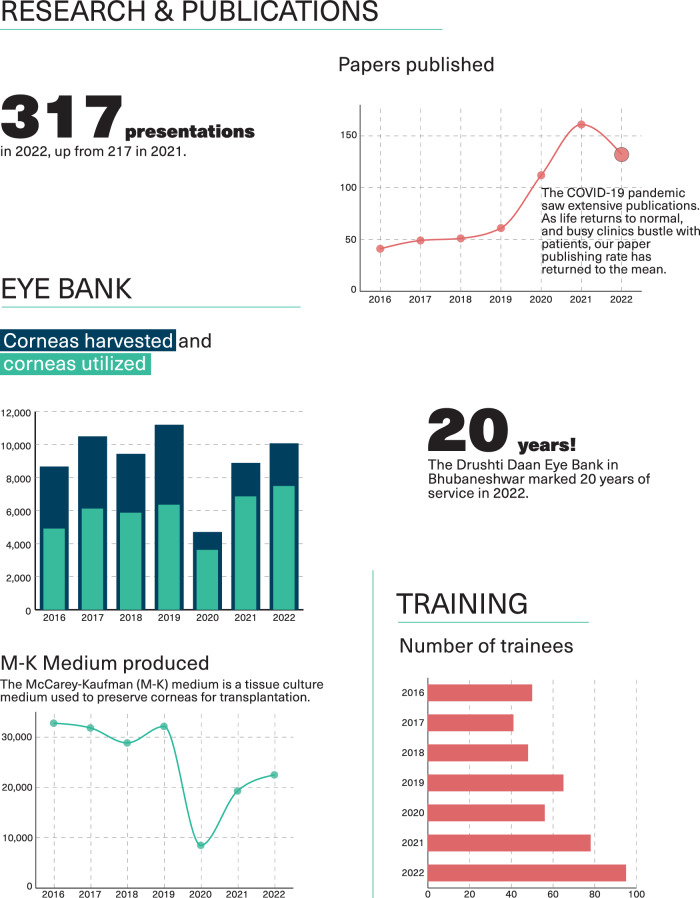
General performance data of LVPEI’s cornea services over 7 years.

The story of LVPEI’s Cornea Care system is deeply entwined with the institute’s story itself. Over these 37 years, this system has evolved into a Cornea Institute—an Institute of Excellence and a Global Resource Centre. In seven years, the Cornea Institute saw 848,000 patients and performed nearly 100,000 surgeries (see Fig. 6). LVPEI’s aspiration of ‘reimagining a relevant and impactful organisation’ applies in great measure to the Cornea Institute. In its work, is the full blossoming of every aspect of care that can improve outcomes for those whose corneas are marred or need repair.

## Summary

### What is known about this topic

- Corneal blindness and vision loss impacts every age group, and the risk factors and the causes are also varied. Preventing, treating, and managing corneal conditions, especially in LMIC contexts, can therefore be quite complex and challenging.

### What this study adds

- The L V Prasad Eye Institute’s cornea care system, developed and refined over four decades in an LMIC context, presents a way forward.
